# Evaluation of an eHealth intervention aiming to promote healthy food habits from infancy -the Norwegian randomized controlled trial *Early Food for Future Health*

**DOI:** 10.1186/s12966-018-0763-4

**Published:** 2019-01-03

**Authors:** Christine Helle, Elisabet R. Hillesund, Andrew K. Wills, Nina C. Øverby

**Affiliations:** 1 Department of Public Health, Sport and Nutrition, Faculty of Health and Sport Sciences, University of Agder, PO Box 422, 4604 Kristiansand, Norway; 2 Faculty of Health Sciences, University of Bristol, Bristol, BS8 1TH UK

**Keywords:** Infant nutrition, Maternal feeding practices, Child eating behavior, Childhood obesity, eHealth, Public health

## Abstract

**Background:**

Strategies to optimize early-life nutrition provide an important opportunity for primary prevention of childhood obesity. Interventions that can be efficiently scaled-up to the magnitude needed for sustainable childhood obesity prevention are needed. The objective of this study was to evaluate the effects of an eHealth intervention on parental feeding practices and infant eating behaviors.

**Methods:**

The Norwegian study *Early Food for Future Health* is a randomized controlled trial. Parents were recruited via social media and child health clinics during spring 2016 when their child was aged 3 to 5 months. In total 718 parents completed a web-based baseline questionnaire at child age 5.5 months. The intervention group had access to a webpage with monthly short video clips addressing specific infant feeding topics and age-appropriate baby food recipes from child age 6 to 12 months. The control group received routine care. The primary outcomes were child eating behaviors, dietary intake, mealtime routines and maternal feeding practices and feeding styles. The secondary outcomes were child anthropometry. This paper reports outcomes at child age 12 months.

**Results:**

More than 80% of the intervention group reported viewing all/most of the video clips addressing infant feeding topics and indicated that the films were well adapted to the child’s age and easy to understand. Children in the intervention group were served vegetables/fruits more frequently (*p* = 0.035) and had tasted a wider variety of vegetables (*p* = 0.015) compared to controls. They were also more likely to eat family breakfast (*p* = 0.035) and dinner (*p* = 0.011) and less likely to be playing or watching TV/tablet during meals (*p* = 0.009) compared to control-group children. We found no group differences for child anthropometry or maternal feeding practices.

**Conclusions:**

Our findings suggest that the eHealth intervention is an appropriate and feasible tool to propagate information on healthy infant feeding to Norwegian mothers. Our study also suggests that anticipatory guidance on early protective feeding practices by such a tool may increase young children’s daily vegetable/fruit intake and promote beneficial mealtime routines.

**Trial registration:**

ISRCTN, ISRCTN13601567. Registered 29 February 2016, http://www.isrctn.com/ISRCTN13601567

**Electronic supplementary material:**

The online version of this article (10.1186/s12966-018-0763-4) contains supplementary material, which is available to authorized users.

## Background

The high worldwide prevalence of childhood overweight and obesity is a huge public health issue [1, 2]. Efficient and easy-to-implement interventions that can be scaled-up to the magnitude needed for sustainable childhood obesity prevention are needed. Since early childhood is a sensitive period for the establishment of dietary habits, strategies to optimize early nutrition could provide an important opportunity for prevention of these conditions. The role of parents in the development of infant food preferences and eating behaviors is essential [3, 4], and makes parents a key target for primary prevention interventions commencing in early childhood.

A recent systematic review including 27 unique randomized controlled trials aiming to reduce the risk of overweight and obesity in infancy and early childhood, concluded that the most promising obesity prevention strategies for children under 2 years of age are those that focus on diet and responsive feeding [5]. Responsive feeding is characterized by caregiver guidance and recognition of the child’s cues of hunger and satiety [6]. A mismatch of caregiver responsiveness to infant-feeding cues is hypothesized to have a role in the development of overweight by impairing the infant’s response to internal states of hunger and satiation [7]. Several previous randomized controlled trials focusing on responsive parenting and feeding have shown a positive impact on child eating outcomes, child growth and child feeding practices [8–13]. In all these trials, the intervention was delivered using traditional approaches (home-visits or face-to-face group settings), which is expensive and hence potentially cost-prohibitive at the level necessary for a national public health strategy. Web-based interventions have the potential to increase parental knowledge and skills while being both easily scalable and accessible. Research on the effectiveness of web-based behavioral change interventions is promising [14], but to our knowledge only very few studies have evaluated the effectiveness of web-based interventions targeted at infant feeding practices and eating behaviors [15, 16].

The *Early Food for Future Health* project started in 2015 with the objective of developing an eHealth intervention that targets beneficial parental feeding practices to promote healthy child eating behavior from infancy. This eHealth intervention draws upon elements from attachment theory, social cognitive theory and the framework of anticipatory guidance to promote knowledge about infant nutrition and facilitate responsive feeding behavior. By using video clips that address age-appropriate infant feeding topics and demonstrate sensitive parent-child interplay in feeding situations together with cooking films showing how to make healthy home-made baby food, the intervention may improve parental feeding practices through social modelling and observational learning and lead to enhanced behavioral capability when it comes to nutritional choices. Details regarding study design and rationale for the intervention content have been previously published [17]. We hypothesized that anticipatory guidance on early feeding practices from child age 6 to 12 months will lead to healthier child eating behaviors and food habits, and more beneficial parental feeding practices compared to usual care. Secondly, we hypothesized that increased use of protective feeding practices will reduce the risk of later childhood overweight and obesity. This paper evaluates these outcomes at child age 12 months.

## Methods

### Study design and intervention components

The study uses the design of a randomized controlled trial to evaluate the results of an eHealth intervention offering anticipatory guidance on infant nutrition and protective feeding behavior. A completed CONSORT checklist is in Additional file 1. The intervention consisted of seven monthly video clips of 3–5 min duration, focusing on feeding-related aspects like appropriate food-types and textures, how taste-preferences evolve and responsive feeding practices; and monthly cooking-films and recipes, demonstrating how to make homemade baby- and family food from easily available ingredients. Parents received an email each month from child age 6 to 12 months with a link to the age-appropriate webpage showing the month’s video clip on the infant feeding topic together with the corresponding recipes and cooking-films. Parents in the control group received routine care from their local child health clinic with regular consultations at child age 6, 8, 10 and 12 months. The consultations are led by a public health nurse and includes measurements of weight and length in combination with conversations related to the child’s health, growth and psychomotor development. At 6 and 12 months the child is also examined by a medical doctor. A TIDieR checklist is presented in Additional file 2.

### Sampling and participants

Parents were eligible to participate in the study if they had a 3–5 months old infant, were literate in Norwegian and responsible for providing food to their infant. Participants were recruited between March and June 2016 via two means – Facebook and child health clinics. We purchased a Facebook advertising package and posted a short video that informed about the study using the University’s Facebook page. The online Facebook’s newsfeed advertisement targeted potential parents aged 18 to 40 years. An email with information about the study was sent to all the Norwegian municipalities´ child health clinics. In total 960 parents signed up on the study’s homepage [18], the majority were mothers (99.6%). From March to September 2016, 718 parents submitted the web-based baseline questionnaire before child age 6 months. Upon receipt of a completed baseline questionnaire, the participant was randomly allocated to either the intervention (*n* = 360) or control group (*n* = 358) based on a computer-generated list using the statistical software SPSS (IBM Corp., Somers; NY, USA). Only the participants’ email-addresses were known when the randomization was administered by the author. Allocation was naturally concealed from recruitment by virtue of the e-recruitment strategy. Follow-up data were collected between Oct 2016 and April 2017. In the absence of response, up to two reminders were sent by e-mail both at baseline and follow-up. The participants had the option both at baseline and follow-up not to answer or complete the web-based questionnaire without having to state any reason, hence we have no data regarding grounds for withdrawal.

### Measurements and outcomes

All socio-demographic and behavioral data were obtained from the study’s web-based, self-administered questionnaires at baseline and follow-up. The study’s primary outcomes were child eating behaviors, dietary intake, mealtime routines and maternal feeding practices and feeding styles. The secondary outcomes were child anthropometry. We also report the participants’ use and experience with the eHealth intervention.

#### The participants’ use and experience with the intervention

In the follow-up questionnaire all participants were asked questions about how they preferred to receive information on infant nutrition *(oral information at the child health clinics, written information/brochures, information on the Internet or information from family/friends)*. Participants had the opportunity to choose more than one option.

Compliance with the intervention was assessed by asking participants if they had seen the films *(all, most, about half, a few, none),* and how many times they had watched each individual film *(0, 1, 2, 3–4 and ≥ 5 times).* They were also asked to what extent they agreed that the content was adapted to the child’s age, easy to understand, that they learned something new, that important topics were raised and whether the films were too long/short *(totally disagree, disagree, neither nor, agree, totally agree, don’t know)*. This information was collapsed into three categories for the analysis: disagree, agree and indifferent *(neither nor/don’t know).*

#### Primary outcomes

##### Child eating behavior

To assess child eating behavior at 12 months of age we used Wardle et al.´s Child Eating Behavior Questionnaire (CEBQ) [19]. This is the most commonly used tool to describe children’s eating behavior. The CEBQ has good internal consistency, test-retest reliability and construct validity [20] and has already been translated to Norwegian [21]. The CEBQ was originally developed for children above 2 years of age but has also been used in children at 12 months of age [22, 23]. A Swedish version of the CEBQ was tested with exploratory factor analysis for use in children between 1 and 6 years of age. Their results supported the use of CEBQ as a psychometric instrument for assessing children’s eating behaviors in children aged 1–6 years [24]. The CEBQ includes 35 items on eating styles, which in the original instrument cluster into eight factors divided into two main dimensions. The Food approach dimension is represented by four factors: *Food responsiveness* (five items, e.g. My child is always asking for food), *Emotional overeating* (four items, e.g. My child eats more when worried), *Enjoyment of food* (four items, e.g. My child loves food) and *Desire to drink* (three items, e.g. My child is always asking for a drink). The Food avoidance dimension is represented by four factors: *Satiety responsiveness* (Five items, e.g. My child gets full up easily), *Slowness in eating* (Four items, e.g. My child eats slowly), *Emotional undereating* (four items, e.g. My child eats less when upset) and *Food fussiness* (Six items, e.g. My child refuses new food at first). Each behavior-item is rated from 1 *(never)* to 5 *(always)* on a five-point Likert scale.

To evaluate the child’s willingness to try new food, we used the Child Food Neophobia Scale (CFNS) modified by Wardle et al. [25]. This is a 6-item version parental report scale based on the original 10-item Food Neophobia Scale developed by Pliner et al. [26]. The CFNS is a reliable and widely used questionnaire which has been validated against behavioral measures of food neophobia [27]. Parents report to what extent they agree in a statement, e.g. “*If my child doesn’t know what’s in a food, s/he won’t try it”* on a four-point scale from 1 *(totally disagree)* to 4 *(totally agree).* The range of possible scores varies between 6 and 24, with a high score indicating high levels of child food neophobia. We used a Norwegian version of the CFNS which has previously been translated and published [28].

##### Child food intake

The infant’s food intake was assessed by a Food Frequency Questionnaire (FFQ) developed for this study. Our questionnaire was based on questionnaires from a Norwegian national dietary survey in 12 months old children [29] and the large population-based Norwegian Mother and Child Cohort Study (MoBa) [30]. The MoBa-study has documented their rationale for use of the FFQ [31], and a Norwegian validation study of the national survey FFQ has been done for child age 24 months [32]. The frequency of intake was assessed without specification of amounts consumed. Questions about drink intake were asked as follows: *“How often does your child drink the following beverages nowadays?”* with answer-options *never/seldom, 1–3 times/week, 4–6 times/week, 1/day, 2/day, 3/day, 4/day* and ≥ *5/day*. Questions about food intake were asked *“How often does your child eat the following food nowadays?”* with answer-options *never/not tried, ‹ 1/week, 1–2/week, 3–4/week, 5–6/week, 1/day, 2/day, 3/day* and *≥ 4/day*. The responses were recoded into a times-per-day score, e.g. *never/seldom* were recoded into zero, *1–3 times/week* into 0.3, *3–4/week* into 0.5, *2/day* into 2.0 etc. For dinner alternatives (see below), the answer-options were recoded into a times-per-week score, e.g. *‹ 1/week* was recoded into 0.5 and *4–6 times/week* was recoded into 5.

We derived two different times-a-day category scores by summing the individual scores in each category: In the *Fruit/vegetable score* we included the times-per-day score for 15 different vegetables/salads *(potato, carrot, rutabaga, sweet potato, cauliflower, broccoli, green salad, spinach, cucumber, tomato, corn, peppers, peas/beans, frozen vegetable mix and salad of mixed raw vegetables)* and 10 different fruits/berries *(orange/clementine, banana, apple, pear, plum, grapes, kiwi, melon, mango, berries - all types).* In the *Non-core food/drink category score* we included the times-per-day score for five snacks *(cakes/cookies or similar, dessert/ice cream, chocolate, sweets, potato chips)* and two sweetened beverages *(lemonade, soda)*.

For dinner-alternatives we made two times-a-week categories: For *Commercially-prepared-dinner score* we summed the individual times-per-week scores for *commercially prepared dinner with vegetables, with meat* and *with fish.* For homemade-dinner score, we summed the individual times-per-week scores for *homemade dinner with vegetables, with meat* and *with fish.*

We computed a key-food variety score to investigate the variation in types of fruits and vegetables that had been offered. “*How often does your child eat the following food (vegetables/fruits/berries) nowadays?”* with answer-options *never/not tried, ‹ 1/week, 1–2/week, 3–4/week, 5–6/week, 1/day, 2/day, 3/day* and *≥ 4/day*, were recoded into tasted and not-tasted. We included 13 vegetables in the vegetable-score and 10 fruits in the fruit score, the same vegetables and fruits as listed above.

#### Child mealtime routines

We included four questions on mealtime routines based upon questions used in the Australian NOURISH study [33]. Parents were asked “How often do the following statements fit the child’s meals today?”, e.g. *My child sits down when having meals*. Response-options were *almost always, often, sometimes, seldom, almost never.* These were recoded into always/often and sometimes/seldom/never.

We further asked how often the child were eating family meals nowadays, specifying “family” to be at least one adult eating the same meal. Response options were *never/seldom, 1 time/week, 2 times/week, 3 times/week, 4 times/week, 5 times/week, every day.* These were recoded into ≤3 times/week and ≥ 4 times/week.

##### Maternal feeding practices

The Infant Feeding Questionnaire (IFQ) [34] was used to assess self-reported maternal feeding practices at baseline and follow-up**.** The original seven-factor validated 20-item questionnaire retrospectively measured feeding practices during the first year of life. Each item is measured on a 5-point Likert scale ranging from 1 *(never)* to 5 *(always),* with mean scores calculated for each factor. The questionnaire was used with minor adaptions in the Australian NOURISH study [35]. In the Australian modified version, the tense was changed from past to present to accommodate concurrent use and it was adapted to the Australian setting with comparatively higher breastfeeding levels. As a result, two of the original factors were excluded, resulting in a five-factor questionnaire. This modified IFQ- questionnaire has previously been used in several Australian studies [36–38]. We found the questionnaire relevant for a Norwegian population where breastfeeding initiation rates are also high compared to other industrialized countries [39]. A translation-back-translation procedure was applied to the IFQ to obtain a Norwegian version. The original English version was translated into Norwegian by one investigator, then translated back into English by another investigator. The English translation was compared with the original IFQ and minor discrepancies were revised based on consensus.

The “Parent provides, child decides”-theme is based on Ellyn Satter’s feeding dynamic approach, in which the caregiver decides *what to eat*, *where to eat* and *when to eat* and allows the child to decide *how much to eat* of the food offered [40, 41]. Parents thus provide the child with opportunities to explore the food while also providing structure and limits. This may be interpreted as an expression of an authoritative feeding style (high in both demandingness and responsiveness), defined as reasonable nutritional demands in conjunction with sensitivity toward the child. Two questions previously used in the NOURISH study [33] assessed this theme: “Who decides *what* the child should eat - you or your child?” and “Who decides *how much* your child should eat - you or your child?”. Answer options were *Only you, Mostly you, You and your child decide equally, Mostly your child, Only your child.* Responses were recoded into *Only/Mostly you* vs. *Decide equally, Mostly your child, Only your child* for deciding *what* the child should eat and *Mostly/Only your child* vs. *Only you, Mostly you, Decide equally* for *how much* the child should eat*.* To fulfill the criterion for the “Parent provides, child decides”-theme, the answers had to be both *Only/Mostly you* for deciding *what* to eat and *Only/Mostly your child* for deciding *how much* to eat.

#### Secondary outcomes

##### Child anthropometric data

Infant weight and length at birth, baseline and follow-up were measured at the child health clinics and reported by the mothers. If the child had not been to the clinic at these time-points, mothers had the opportunity to omit responding. Z-scores for weight-for-age (WAZ) and BMI-for-age (BMIZ) at birth, baseline and follow-up were calculated using the software program WHO Anthro version 3.2.2. (Department of Nutrition, World Health Organization, Geneva, Switzerland) and macros.

### Statistics

Sample size was estimated based on the Australian NOURISH study, which used a similar population and assessed similar primary and secondary outcomes [33]. They estimated that 265 participants per group would be sufficient to detect clinically meaningful differences at the study end (child age 22–25 months) for the prevalence of intake of key foods indicative of dietary quality and key parent practices supporting self-regulation, and they aimed to recruit 820 participants assuming a 65% completion rate. However, their observed differences in weight measures lacked power [10]. At child age 14 months, the NOURISH reported findings for “Maternal feeding practices”, measured by the Infant Feeding Questionnaire (IFQ), as in our study [35]. Their observed differences were small and only reached significance for one subscale (*Awareness of infant satiety and hunger cues;* 4.2 versus 4.1 for intervention versus control, respectively; SD 0.5 for both groups). We considered these effect sizes shown in NOURISH to be meaningful since we are evaluating a public health intervention with the potential to reach a large population. Taking this into account, we estimated that we would need approximately 400 participants in both control and intervention arm and aimed to recruit 500 in each group to be able to detect differences in the primary outcome maternal feeding practices (IFQ) at child age 12 months with 80% power and type error I of 5%, assuming a completion rate of 80%.

#### Factor analysis

Confirmatory factor analysis was performed to analyze the underlying structure of the CEBQ, resulting in the same eight factors as in the original CEBQ. The resulting values for Cronbach’s α varied between 0.7 and 0.9, indicating a moderate to good internal reliability of the CEBQ-subscales in this sample (Additional file 3). We performed a confirmatory factor analysis also for the IFQ*.* The factor analysis in resulted in the same five factors as in the modified Australian version. Cronbach’s α ranged from 0.6–0.8, indicating a moderate to good internal reliability of the subscales in this cohort, except from the scale *Feeding on a schedule* (Cronbach’s α = 0.5) (Additional file 4). For the CFNS, Cronbach’s α was 0.89, indicating a good internal consistency of the scale in this sample.

#### Between-group analyses

All analyses were conducted on an intention-to-treat basis. Characteristics of the groups at baseline were described using descriptive statistics. Linear regression was used to estimate differences between the control and intervention group for *Child eating behavior* and *Child food intake*. Multiple linear regression was used to estimate differences in *Maternal feeding practices* (measured by the IFQ), adjusting for baseline differences in the IFQ-scores. Odds ratios were used as a measure of effect for the dichotomous outcomes in *Selected mealtime routines* and were estimated using logistic regression. For *Child anthropometric outcomes*, the group means were compared first without adjusting and second adjusting for baseline values using linear regression. Lastly; since higher scores on the *Food-approaching dimension score* (CEBQ) has been associated with increased weight gain in children, we explored the relationship between the *Food-approaching dimension score* and BMI-for-age z-score at 12 months by multiple linear regression, adjusting for group-status and baseline BMI-for-age z-score.

#### Sensitivity analysis

A small minority of the participants in the intervention group reported not engaging with the intervention (8% for infant feeding videos, 20% for cooking films). A sensitivity analysis was performed by excluding the non-adherers in the intervention group (*n* = 22) who reported to have seen none/a few of the infant feeding videos for selected primary outcomes *(Maternal feeding practices*, *Child eating behavior* and *Child food-category intake)*.

Statistical significance was defined as a *p*-value less than .05, and all tests of the intervention were 2-sided. The data were analyzed by using IBM SPSS Statistics version 4.0 (IBM Corp., Somers; NY, USA).

## Results

### Study sample

**Fig. 1:**
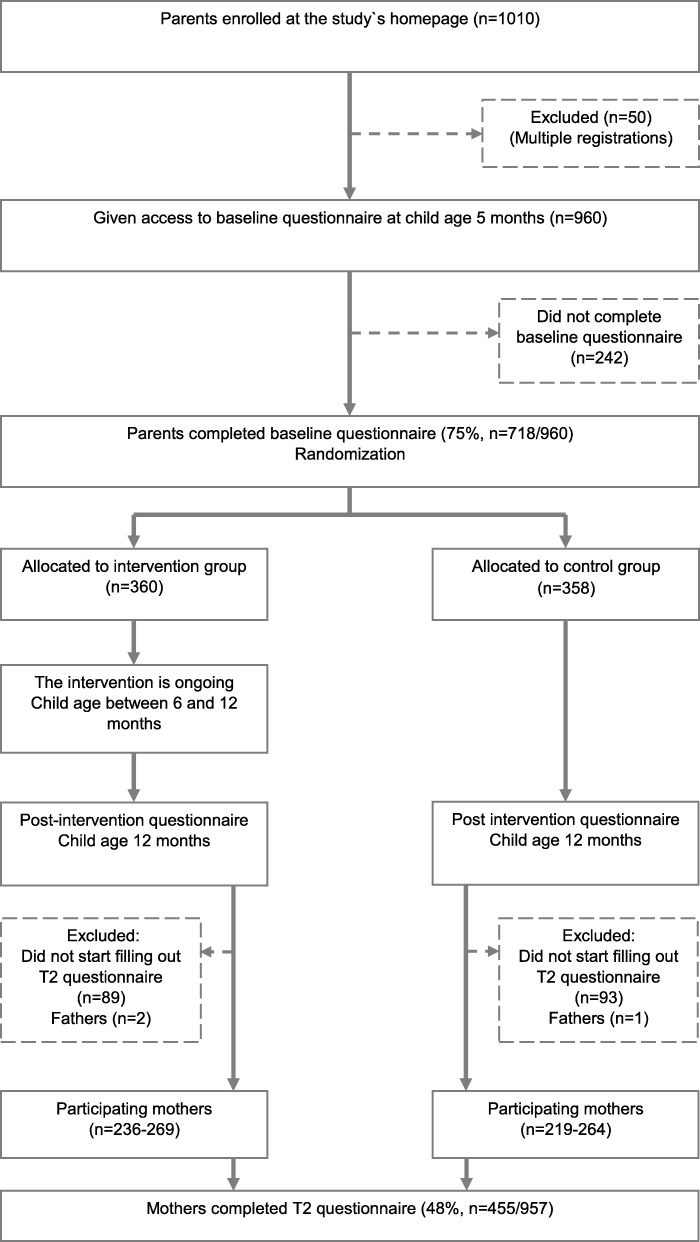
Flow diagram for the *Early Food for Future Health* study

The flow of participants through the study is presented in Fig. 1. From the 957 eligible participants recruited, 715 (75%) completed the baseline questionnaire at child age 5.5 months and were randomized to either control or intervention group. At 12 months of age, 455/715 (63%) mothers completed the follow-up questionnaire. Of these, 236 mothers had been allocated to the intervention group and 219 allocated to the control group. The average age of the child upon submission of the follow-up questionnaire was 12.1 ± 0.3 months. We included all available responses for each of the outcome variables, making the total number of participants in the analyses vary between 236 and 269 in the intervention group and 219 and 264 in the control group.

Descriptive baseline characteristics of participants who were lost to follow-up and participants who retained in the study (analysis sample) showed that mothers lost to follow-up were on average significantly younger (29.8 years versus 30.8 years; *p* = 0.008) and less likely to have a higher education (77% versus 84%; *p* = 0.016). Furthermore, we explored the data for potential differences between mothers allocated to the control group and mothers allocated to the intervention group within the group lost to follow-up. There was no evidence of differences across the maternal and infant characteristics between groups (Additional file 5).

Baseline maternal and child characteristics for this study are presented by group status in Table 1. The groups were well balanced across the range of maternal and infant characteristics, indicating that the randomization was successful.

**Table 1:** Baseline characteristics of mothers/infants allocated to the control group compared with the intervention group

Variable	Control (*n* = 264)^a^ % (count) or mean ± SD	Intervention (*n* = 269)^b^ % (count) or mean ± SD	Total (*n* = 533)^c^ % (count) or mean ± SD
Mother			
Age (years)	30.2 ± 4.1	30.9 ± 4.4	30.6 ± 4.3
Not Norwegian as native language	7.6 (20)	5.9 (16)	6.8 (36)
First-time mother (for infant participating in the survey)	59.5 (157)	57.2 (154)	58.3 (311)
Marital status			
Married	35.6 (94)	44.6 (120)	40.2 (214)
Cohabitant	62.1 (164)	52.8 (142)	57.4 (306)
Not married/cohabitant	2.3 (6)	2.6 (7)	2.4 (13)
Education (College/university degree)	82.0 (214)	84.6 (225)	83.3 (439)
Main activity (before pregnancy)			
Working fulltime	81.4 (214)	81.6 (217)	81.5 (431)
Working part time	6.1 (16)	6.4 (17)	6.2 (33)
Student	6.8 (18)	7.5 (20)	7.2 (38)
Other/Not working	5.7 (15)	4.5 (12)	5.1 (27)
BMI (kg/m^b^)	24.9 ± 4.2	24.9 ± 4.5	24.9 ± 4.4
Smoking	3.8 (10)	3.3 (9)	3.6 (19)
Use of snus	4.9 (13)	5.2 (14)	5.1 (27)
Infant			
Gender (female)	50.4 (133)	49.6 (133)	49.9 (266)
Gestational age > 38 weeks	91.3 (241)	91.1 (245)	91.2 (486)
Birth weight (g)	3586 ± 513	3557 ± 472	3571 ± 492
Weight at 5 months (g)	7656 ± 924	7503 ± 887	7577 ± 907
Exclusive breastfed first month	67.4 (178)	70.3 (189)	68.9 (367)
Introduced to solid food before 4 months of age	6.1 (16)	4.1 (11)	5.1 (27)

^a^ The total number of participants in the analyses vary between 219 and 264 because of missing data for some variables

^b^ The total number of participants in the analyses vary between 236 and 269 because of missing data for some variables

^c^ The total number of participants in the analyses vary between 455 and 533 because of missing data for some variables

### Participants´ use and experience with the intervention

Almost 80% of the mothers reported that they preferred to source information on infant nutrition from the Internet, and 67% stated as important/very important that this information came from public authorities. The second most preferred source was books or brochures (71%), followed by oral communication from public health nurses at the child health clinics (44.5%) and information from friends and family 31.5%).

**Fig. 2:**
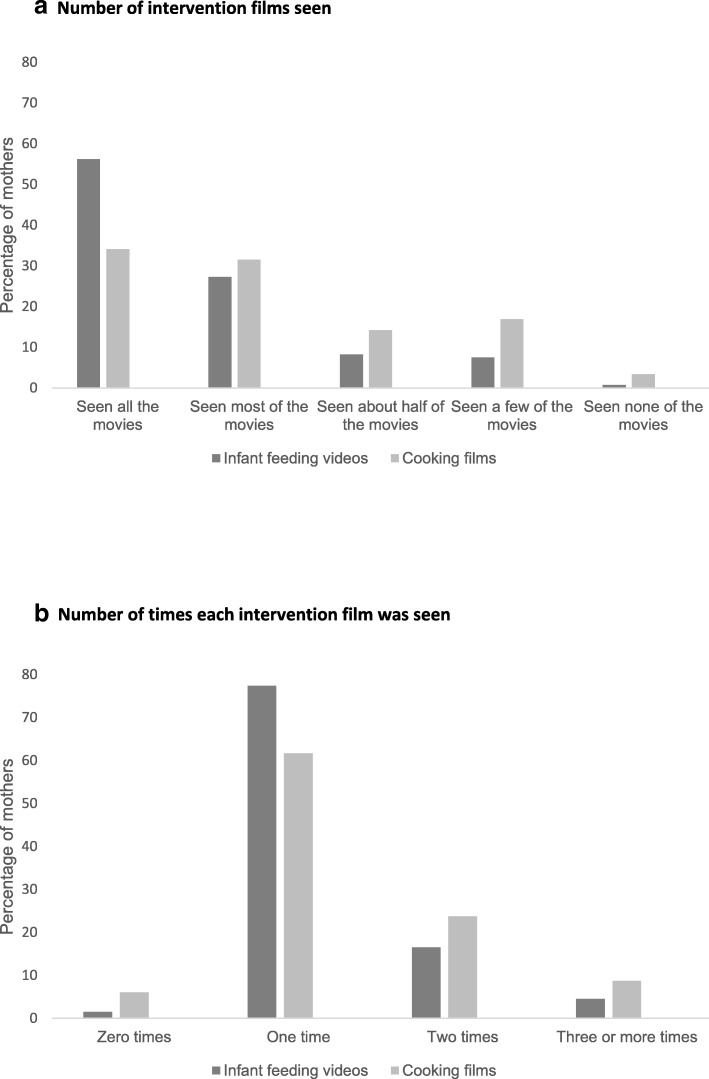
Participants’ reported use of the intervention: **a** Number of intervention film seen; **b** Number of times each intervention film was seen

In the intervention group, 85% of the mothers reported watching all or most of the video clips addressing infant feeding topics and 66% reported watching all or most of the cooking films. A total of 8% of the mothers reported that they had watched none or only a few of the feeding videos, and 20% reported watching none or a few of the cooking-films. Most mothers reported that they had watched the infant feeding- and cooking-film once (78 and 62% respectively). Only a small proportion reported that they had watched the infant feeding- or cooking-films three times or more (5 and 9% respectively) (Fig. 2). When comparing the mothers with high vs. low education (college/university degree vs. not), we found no evidence of a difference for seeing all or most of the films (83% within the high education group vs. 87% within the low education group; *p* = 0.49).

**Fig. 3:**
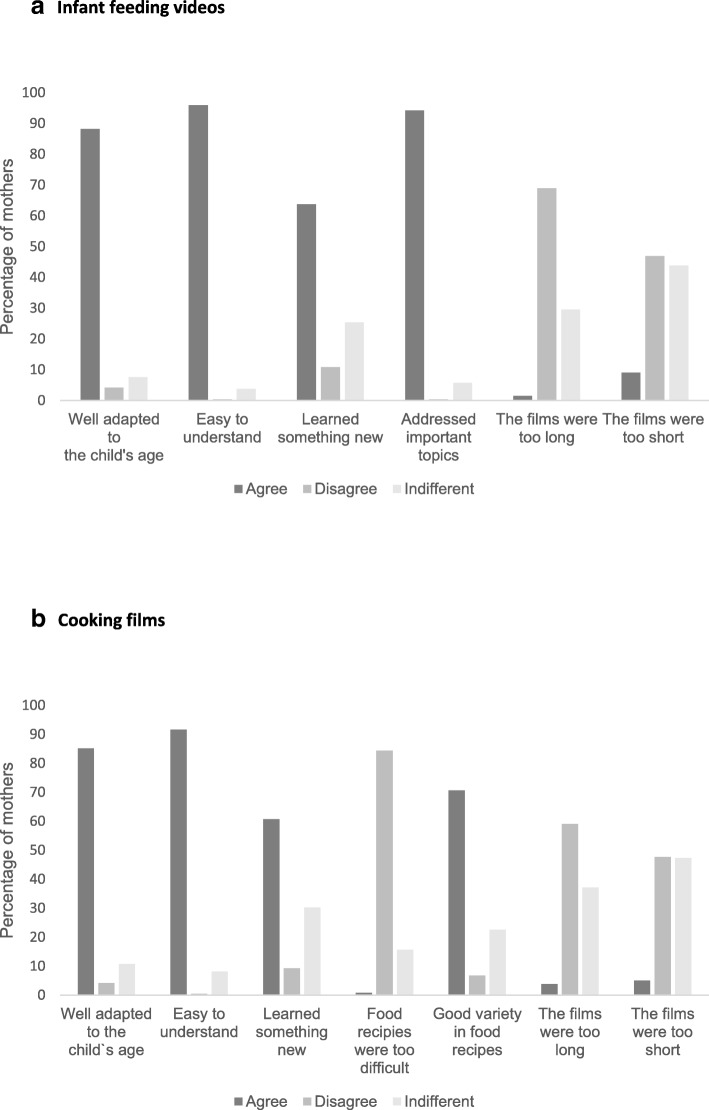
Participants’ reported experience with the intervention: **a** Infant feeding videos; **b** Cooking films Answer-options were given on a 6-point Likert-scale: *highly disagree, disagree, neither-nor, agree, highly agree, don’t know* and recoded into *agree, disagree* and *indifferent (neither-nor/don’t know)*

The majority of intervention participants reported that the video clips addressing infant feeding topics were well adapted to the child’s age (88%), easy to understand (96%) and addressed important topics (94%) (Fig. 3). A minor proportion reported that they hadn’t learned something new (11%). For the cooking-films, the majority found the recipes well adapted to the child’s age (85%), easy to understand (92%) and of good variety (71%). A minority reported that they had not learned something new (9%). For both the infant feeding- and cooking-films, less than 10% reported that the films were of too short or too long duration. When comparing the mothers with high vs. low education (college/university degree vs. not), we found no evidence of a difference for reporting that the films were easy to understand (96% within the high education group vs 97% within the low education group; *p* = 0.60).

### Primary outcomes

#### Child eating behavior, food intake and mealtime routines

Table 2 shows the means and differences in means for *Child eating behavior*, *Food-category intake* and *Key-food variety-scores* at 12 months of age. There was a group difference for the subscale *Food Responsiveness* (*p* = 0.01) and for the CEBQ’s *Food approach dimension* (*p* = 0.002), where intervention group children had higher scores on both scales. We found no group difference in willingness to try new food assessed by the *Child Food Neophobia Scale* (CFNS).

**Table 2:** Child eating behavior, food-category intake and key-food variety-scores in the control and intervention group at child age 12 months

Item	Control mean ± SD (*n* = 264)^a^	Intervention mean ± SD (*n* = 269)^b^	Difference (95% CI)	*p* value
*Child Eating Behavior Questionnaire* ^*c*^				
Food responsiveness	2.28 ± 0.69	2.44 ± 0.71	0.17 (0.04 to 0.30)	0.010
Emotional over-eating	1.41 ± 0.49	1.48 ± 0.51	0.07 (− 0.02 to 0.16)	0.15
Enjoyment of food	3.99 ± 0.62	4.09 ± 0.63	0.10 (− 0.01 to 0.21)	0.086
Desire to drink	2.09 ± 0.86	2.25 ± 0.88	0.16 (− 0.00 to 0.32)	0.051
Satiety responsiveness	2.85 ± 0.59	2.78 ± 0.62	− 0.07 (− 0.18 to 0.43)	0.23
Slowness in eating	2.67 ± 0.61	2.65 ± 0.73	− 0,02 (− 0.14 to 0.10)	0.74
Emotional under-eating	3.29 ± 0.82	3.27 ± 0.78	− 0.02 (− 0.17 to 0.13)	0.79
Food fussiness	1.87 ± 0.72	1.87 ± 0.66	0.00 (− 0.13 to 0.13)	0.98
CEBQ; Food approach dimension	2.44 ± 0.44	2.56 ± 0.44	0.12 (0.04 to 0.20)	0.002
CEBQ; Food avoidance dimension	2.67 ± 0.43	2.64 ± 0.46	−0.2 (− 0.11 to 0.06)	0.56
*Child Food Neophobia Scale* ^*d*^	9.50 ± 3.91	9.07 ± 3.34	− 0.43 (− 1.09 to 0.23)	0.20
*Child food intake, categories* ^*e*^				
Fruit/vegetables*, times-per-day score*	5.93 ± 2.44	6.44 ± 2.77	0.51 (0.04 to 0.98)	0.035
Non-core foods/drinks; *times-per-day score*	0.24 ± 0.23	0.22 ± 0.21	− 0.02 (− 0.06 to 0.02)	0.42
Commercially prepared dinner; *times-per-week score*	1.20 ± 1.84	1.00 ± 1.48	− 0.20 (− 0.50 to 0,10)	0.19
Homemade dinner; *times-per-week score*	2.54 ± 1.80	2.76 ± 2.68	0.23 (− 0.19 to 0.64)	0.28
*Key-food variety-score; number of units tasted* ^*f*^				
Vegetables *(n*_*tot*_ *= 13)*	10.62 ± 2.03	11.05 ± 1.77	0.43 (0.08 to 0.77)	0.015
Fruit *(n*_*tot*_ *= 10)*	8.41 ± 1.80	8.58 ± 1.57	0.17 (− 0.13 to 0.48)	0.26

Linear regressions are used for between-group comparison of continuous outcomes

^a^ The total number of participants in the analyses vary between 219 and 264 depending on the outcome

^b^ The total number of participants in the analyses vary between 236 and 269 depending on the outcome

^c^ Behavior items rated from 1 *(never)* to 5 *(always)* on a five-point Likert scale

^d^ The range of possible responses varies between 6 and 24, with a high score indicating high levels of child food neophobia

^e^ Answer-options *never/not tried, ‹ 1/week, 1–2/week, 3–4/week, 5–6/week, 1/day, 2/day, 3/day* and *≥ 4/day* recoded into a *times-per-day/times per week* score

^f^ Answer-options *never/not tried, ‹ 1/week, 1–2/week, 3–4/week, 5–6/week, 1/day, 2/day, 3/day* and *≥ 4/day* dichotomized into *tasted/not tasted*

Children in the intervention group had a higher times-per-day score for *fruits/vegetables* than children in the control group (*p* = 0.035). For number of fruits and vegetables tasted, there was a group difference for vegetables (*p* = 0.015), where children in the intervention group had a higher score.

Table 3 shows child mealtime routines at 12 months of age in the control and intervention group. Children in the intervention group were more likely to eat the same for dinner as the rest of the family (*p* = 0.004) and less likely to eat a separate made dinner (*p* = 0.006) and to be playing or watching TV/tablet during meals (*p* = 0.009) compared to children in the control group. There was no evidence of a difference between the groups for child sitting at the dinner table while eating. Further, children in the intervention group were more likely to eat breakfast (*p* = 0.035) and dinner (*p* = 0.011) together with their family compared to children in the control group. There was no evidence for a difference between groups with respect to eating lunch or supper together.

**Table 3:** Child mealtime routines at 12 months in the control and intervention group

Item	Control % (count) (*n* = 264)^a^	Intervention % (count) (*n* = 269)^b^	OR^c^	CI 95%	*p* value
*Mealtime habits* ^*d*^					
Child eating the same for dinner as the rest of the family	68.8 (161)	80.3 (196)	1.85	1.22–2.82	0.004
Parents making separate dinner for the child	27.8 (65)	17.2 (42)	0.54	0.35–0.84	0.006
Child is sitting at the dinner table when eating	98.7 (231)	99.6 (244)	3.16	0.37–30.58	0.32
Child playing or watching TV / tablet while eating	8.1 (19)	2.5 (6)	0.29	0.11–0.73	0.009
*Child eating meal together with family* ^*e*^ *≥4 times per week*					
Breakfast	67.5 (186)	76.2 (158)	1.54	1.03–2.31	0.035
Lunch	48.3 (113)	45.5 (111)	0.89	0.62–1.28	0.54
Dinner	84.6 (225)	92.2 (198)	2.15	1.20–3.88	0.011
Supper	32.9 (77)	38.5 (94)	1.28	0.88–1.86	0.20

Logistic regression is used for between-group comparison of dichotomous outcomes

^a^ The total number of participants in the analyses vary between 219 and 264 depending on the outcome

^b^ The total number of participants in the analyses vary between 236 and 269 depending on the outcome

^c^ Control group as reference group

^d^*Almost always/often* reported

^e^*Family* defined as at least one adult eating the same meal

#### Maternal feeding practices

Table 4 presents maternal feeding practices assessed by *the Infant Feeding Questionnaire* at child age 12 months. There was no between-group difference for any of the subscales. Also, for the *Parent provide-child decide theme,* we found no significant difference between the intervention and control group for mostly/only the mother to decide *what* the child should eat and mostly/only the child to decide *how much* to eat (55% vs. 47% respectively, *p* = 0.079).

**Table 4:** Maternal feeding practices in the control and intervention group at child age 12 months

Item	Control mean ± SD (*n* = 264)^a^	Intervention mean ± SD (*n* = 269)^b^	Adjusted Mean Difference^c^(95% CI)	*P* value
*Infant feeding Questionnaire (IFQ)* ^*d*^				
Awareness of infant satiety and hunger cues	4.14 ± 0.61	4.08 ± 0.60	− 0.02 (− 0.12 to 0.08)	0.65
Using food to calm fussiness	2.10 ± 0.70	2.13 ± 0.68	0.03 (− 0.09 to 0.15)	0.61
Feeding on schedule	3.03 ± 0.78	3.05 ± 0.75	0.003 (− 0.13 to 0.14)	0.96
Concern about infant under-eating and being underweight	1.90 ± 0.81	1.84 ± 0.76	− 0.09 (0.28 to 0.48)	0.18
Concern about infant over-eating and being overweight	1.53 ± 0.64	1.56 ± 0.66	0.02 (− 0.08 to 0.13)	0.68

Multiple linear regressions are used for between-group comparison of continuous outcomes

^a^ The total number of participants in the analyses vary between 219 and 264 depending on the outcome

^b^ The total number of participants in the analyses vary between 236 and 269 depending on the outcome

^c^ Adjusted for the respective IFQ baseline-scores

^d^ IFQ: 5-point likert-style scale (*1 = never, 2 = rarely, 3 = sometimes, 4 = often, 5 = always)*

### Secondary outcomes

#### Child anthropometric outcomes

Table 5 shows the means and differences in means for child anthropometry at baseline and 12 months. There was no evidence of a between-group difference at 12 months of age.

**Table 5:** Anthropometric data (mean ± SD) for control and intervention group at baseline (5.5 months) and follow-up (12 months)

Group Allocation	Unadjusted			Adjusted for baseline values	
	Mean ± SD	Mean difference (95% CI)	*p* value	Mean difference^c^(95% CI)	*p* value
Child BMI					
Baseline^a^					
Intervention	17.04 ± 1.46	− 0.26 (− 0.56 to 0.42)	0.091		
Control	17.30 ± 1.47	NA			
Follow-up^b^					
Intervention	17.10 ± 1.40	−0.01 (− 0.28 to 0.26)	0.96	0.16 (− 0.09 to 0.42)	0.21
Control	17.11 ± 1.31	NA			
BMI z-scores					
Baseline					
Intervention	−0.06 ± 0.95	−0.17 (− 0.37 to 0.03)	0.095		
Control	0.11 ± 0.97	NA			
Follow-up					
Intervention	0.31 ± 0.99	−0.02 (−0.21 to 0.17)	0.85	0.09 (−0.09 to 0.27)	0.30
Control	0.33 ± 0.88	NA		NA	
Change in BMI z-scores					
Baseline to follow-up					
Intervention	0.30 ± 0.85	0.17 (−0.03 to 0.37)	0.097	0.15 (−0.04 to 0.34)	0.13
Control	0.14 ± 0.77	NA		NA	

Linear regression and multiple linear regression are used for between-group comparison of continuous outcomes

^a^ Baseline: *n* = 365

^b^ Follow-up: *n* = 380

^c^ Adjusted for the respective baseline value

#### Relationship between the CEBQ’s food approach dimension and infant weight gain

We explored the relationship between the CEBQ’s *Food approach dimension score* and BMI-for-age z-score at child age 12 months by multiple regression, adjusting for group status and baseline BMI-for-age z-score. The CEBQ’s *Food approach dimension score* was a significant independent predictor of BMI-for-age z-score at child age 12 months (β = 0.12, *p* = 0.014), while group allocation was not (β = 0.037 (intervention-group), *p* = 0.45). Baseline BMI-for-age z-score was a strong independent predictor (β = 0.62, *p* < .01).

### Sensitivity analysis

A sensitivity analysis was performed by excluding the non-adherers in the intervention group for selected primary outcomes. The findings were quantitatively similar except for a small increase of precision (results available on request).

## Discussion

This paper reports the results of an eHealth intervention aiming to promote healthy food habits from infancy. Outcomes reported within a month after the intervention was completed, provides promising evidence that online anticipatory parental guidance on protective feeding practices may result in higher daily vegetable/fruit intake and more beneficial mealtime routines. The intervention did not impact child anthropometric outcomes. To our knowledge, *Early Food for Future Health* is one of the very first studies to investigate and report data on whether beneficial parental feeding practices and healthy child eating habits can be promoted from infancy by use of an Internet-based approach.

### Participant’s use and experience with the intervention

The majority of today’s parents search for parental-related information on the Internet. A literature review on parenthood, information and support on the Internet found that although first-time middleclass mothers aged 30–35 years were the most frequent users, several of the included studies reported reduced socioeconomic class differences on parental websites [42]. Parents most often seek online information regarding child health care needs [43]. In our study almost 80% of the participating mothers reported the Internet as their preferred source of information on infant nutrition. The study’s high proportion of well-educated mothers in their early thirties may have affected these numbers.

In Norway, social differences in health are more prominent than in many other European countries [44]. Reducing socioeconomic inequalities in health is an important public health goal, and care should be taken for interventions to be adapted to groups with lower socioeconomic status. In the present study more than 80% of the mothers reported viewing all or most of the intervention’s video clips addressing infant feeding topics. A similar high proportion of participants agreed that the films were well adapted to the child’s age and easy to understand. These figures suggest that the majority of participants experienced the intervention as relevant and feasible. We found no differences between mothers with higher and lower education for seeing all or most of the movies or agreeing that the intervention-films were easy to understand. Lower parental literacy and numeracy skills are associated with poorer understanding and reduced application of health information in the care of their children [45] and with worse child health outcomes [46, 47]. This also applies to childhood obesity [48]. The use of videos instead of written material in the present study may have been beneficial for the mothers in the lower-education group [49] and contributed to our findings.

### Child eating behavior, food-intake and mealtime routines

The sub-scale scores for the *Child Eating Behavior Questionnaire* in our sample were comparable to previous findings for the same age group [24]. For *Food responsiveness* and the *Food approach dimension*, we found higher scores for the intervention group. Food-approaching behavior has previously been associated with increased food intake and larger weight gain in children [50, 51]. Higher values of the *Food approach dimension* could therefore be interpreted as adverse or contradictive findings. When exploring this relationship further in our sample, we found that the *Food-approaching dimension score* was an independent positive predictor for BMI-for-age z-score at 12 months while group affiliation had no significant influence. It will be important to investigate this further in subsequent follow-up studies.

To date there are few reported studies of Internet-based interventions on children’s intake of fruits and vegetables. A recently published study, the Growing Healthy mHealth program (*n* = 645) reports the effects of a mobile-phone health intervention on parental feeding practices, infant food preferences, and infant satiety responsiveness [52]. They found no effect on infants’ core and noncore food exposure at 9 months of age. A Swedish study (*n* = 315) assessed the effectiveness of a 6-month mHealth obesity prevention program in healthy children aged 4.5 years. They found no significant differences between intervention and control group for intakes of fruits, vegetables, candy, and sweetened beverages [53]. A recently published Cochrane review on interventions for increasing fruit and vegetable consumption in children ≤5 years including 50 trials (*n* = 10,267), concluded that the evidence for effective interventions to increase eating of fruit and vegetables remain sparse. Child feeding interventions appeared to increase vegetable intake by children, but this conclusion was based on very low-quality evidence, the effect sizes were small, and long-term follow-up was required [54]. In our study children in the intervention group had a higher *times-per-day* score for servings of fruit/vegetables and for number of vegetables tasted. To our knowledge, the current study is one of few to report results of a web-based intervention aiming to promote healthier food habits from infancy. Our findings contribute to the existing body of research on how fruit and vegetable intake in early childhood can be modified.

Eating meals together as a family has proven to be beneficial for many aspects of child and adolescent health, including better dietary intake and lower risk of childhood obesity [55–57]. On the contrary, eating whilst watching television has been negatively associated with diet quality among children and the emotional atmosphere of the meal [58, 59]. In our study, children in the intervention group were more likely to eat breakfast and dinner together with their family compared to children in the control group. Children in the control group were more likely to eat a separately made dinner and to be playing or watching TV/tablet during meals compared to children in the intervention group. Dietary behaviors are established during infancy [60] and tracks from childhood to adulthood [61]. Establishing healthy eating habits and beneficial mealtime routines from early on is therefore important also in long term. Our results suggest that the intervention was successful in promoting early beneficial mealtime routines that can provide a good foundation for further growth, development and health.

We found no differences between the groups for food neophobia assessed by the *Child Food Neophobia Scale.* Refusal of unfamiliar food begins in infancy but is more common in toddlers and peaks between 2 and 6 years of age [62]. It may therefore be that mapping food neophobia at child age 12 months is somewhat early in terms of normal eating development and therefore should be further explored in subsequent follow-up studies.

### Maternal feeding practices

To recognize and respond appropriately to the infant’s cues of hunger and satiety is critical in supporting the infant’s innate capacity to self-regulate food intake [7, 35]. When designing the intervention, emphasis was placed on the importance of a responsive feeding style. Despite this, we found no group differences for any of the subscales in the Infant Feeding Questionnaire. Based on comparison of the five subscales, the mean scores in our sample were similar to what they found in the NOURISH study at child age 14 months, although they found a small but significant group-difference for one of the subscales [35]. Our results are also in line with the Growing Healthy study, in which there were no significant between-group differences for any of the IFQ’s subscales at child age 9 months [52]. Whether the intervention’s delivery mode has importance for influencing feeding practices remains unclear. Studies addressing early parental feeding practices have heterogenous study designs which makes direct comparisons difficult. However, videotapes of interactions between parent and child have previously been found to improve parent-child interplay and attachment security [63, 64]. This makes it likely that web-based videos also have potential to improve parent-child interactions related to feeding. As in the NOURISH study, the mean score for *Concern about underweight* was higher than the mean score for *Concern about overweight*. This may indicate that cultural norms and perceptions of infant weight have not changed in line with the increasing incidence of childhood overweight and obesity. A qualitative systematic review of maternal infant feeding practices in the weaning period describe how a higher infant weigh is seen as a “safety net” and interpreted as a sign of good mothering in developed countries [65].

A higher proportion of mothers in the intervention group (55% vs. 47%) reported that mostly/only the mother decided *what* to eat and mostly/only the child *how much* to eat. This may suggest that more mothers in the intervention group tend to use a feeding style characterized by an ability to both enforce rules as well as being sensitive to the child’s needs. However, this difference did not reach significance.

### Child anthropometry

The intervention had no impact on child anthropometric outcomes at child age 12 months. As this was a universal primary prevention intervention, irrespective of maternal- or child obesity risk, we focused on promoting healthy growth rather than on preventing obesity. This may explain our results. However, the establishment of healthy eating habits from infancy may affect the child’s weight development in the longer term. Potential long-term effects on weight will therefore be investigated in subsequent follow-up studies.

### Strengths and limitations

The study’s strengths are the randomized controlled trial design and a participant group consisting of both first-time mothers and mothers with older children. Similar studies have largely included only first-time mothers [33, 66]. Mothers with older children may already have developed habits related to infant feeding and family food. As it is more difficult to change established behaviors, our findings are likely to be conservative. Larger studies are needed to examine if effects differ by number of children. The public health utility of the intervention is important to acknowledge. This is an easily accessible, feasible, online intervention with the potential to be both scalable and cost-effective.

There are also some limitations of our study. Certain comparisons may lack power. Despite a comparatively large number of participants, the retention rate was somewhat lower than expected. A total of 1010 participants registered themselves on the study’s homepage, but the actual number turned out to be 960 because of multiple registrations. In addition, more participants than expected dropped out between registration and baseline and between baseline and follow-up. However, the dropout appears to be balanced between the intervention and the control group, so a mechanism for introducing bias is difficult to conceive. The mothers in the present study volunteered for participating, which in epidemiological research is known to lead to a more well-educated study-sample [67, 68] as seen in our study. A high proportion of participants had education at college or university level (83%) compared to national data for women in the same age-group (58%) [69]. Besides, a small proportion of participants did not have Norwegian as native language (7%), considering that 17% of Norway’s population has an immigrant background [70]. Larger studies are desirable to examine if effects differ by maternal education or ethnic background. The use of self-reported data may reduce reliability and the nature of the sample may have implications for the generalizability of our findings. Finally, the assessment of study compliance was based on self-report and thus reliant on the participants’ memory. This may be a potential source of recall bias. Further, there may also be a possibility of over-reporting based on social desirability bias in the intervention group.

## Conclusion

To date, there has been limited number of studies reporting on eHealth interventions commencing in infancy, addressing modifiable early risk factors associated with later childhood obesity. To the best of our knowledge, this paper is one of few to report results of a web-based intervention aiming to promote healthy food habits from infancy. Further, very few interventions can easily be scaled-up to the magnitude needed for sustainable childhood obesity prevention. This study provides promising evidence that an Internet-based intervention providing parental anticipatory guidance on early protective feeding practices can increase daily vegetable/fruit intake and promote more beneficial mealtime routines. For weight-related outcomes there were no between-group differences. Future follow-up studies are important for the evaluation of longer-term effects.

## Additional files

Additional file 1: CONSORT Extension for within person trials checklist. (PDF 199 kb)

Additional file 2: Template for Intervention Description and Replication (TIDieR) checklist for *Early Food for Future Health. (PDF 240 kb)*

Additional file 3: Confirmatory factor analysis for the Child Eating Behavior Questionnaire; factor loadings for all items and Cronbach alpha scores for each factor structure. (PDF 210 kb)

Additional file 4: Confirmatory factor analysis for the Infant Feeding Questionnaire; factor loadings for all items and Cronbach alpha scores for each factor structure. (PDF 194 kb)

Additional file 5: Group comparisons of baseline characteristics between participants who retained in the study and those who were lost to follow-up, and between the control and intervention group within the group of participants lost to follow-up. (PDF 95 kb)

## Abbreviations

CEBQ: Child Eating Behavior Questionnaire
CFNS: Child Food Neophobia Scale
ICT: Information and Communication Technology
IFQ: Infant Feeding Questionnaire
NCD: Non-Communicable Diseases
RCT: Randomized Controlled Trial
