# Supplementary material for: Evaluation of an eHealth intervention aiming to promote healthy food habits from infancy -the Norwegian randomized controlled trial Early Food for Future Health
Source: Int J Behav Nutr Phys Act. 2019 Jan 3;16:1. doi: 10.1186/s12966-018-0763-4 (PMC6318886; doi:10.1186/s12966-018-0763-4)
Supplement: Supplementary file 3 — Confirmatory factor analysis for the Child Eating Behavior Questionnaire; factor loadings for all items and Cronbach alpha scores for each factor structure. (PDF 210 kb) [file 12966_2018_763_MOESM3_ESM.pdf]

Supplemental Table1: Factor loadings for all items of the Child Eating Behavior Questionnaire (CEBQ) and Cronbach alpha scores for each factor structure

Items	Factors determined through factor analysis [a]								Original scale
	Food responsiveness FR	Emotional over-eating EOE	Enjoyment of food EF	Desire to drink DD	Satiety Responsiveness SR	Slowness in eating SE	Emotional under-eating EUE	Food fussiness FFF	
My child is always asking for food			.478						FR
If allowed to, my child would eat too much	**.595**		.346						FR
Given the choice, my child would eat most of the time	**.704**		.384						FR
Even if my child is full up s/he finds room to eat his/her favourite food	**.668**								FR
If given the chance, my child would always have food in his/her mouth	**.735**								FR
My child eats more when worried		**.758**							EOE
My child eats more when annoyed		**.802**							EOE
My child eats more when anxious		**.827**							EOE
My child eats more when s/he has nothing else to do	.552								EOE
My child loves food			**.722**					-.321	EF
My child is interested in food			**.639**					-.360	EF
My child looks forward to mealtimes			**.679**						EF

My child enjoys eating			**.699**					-3.90	EF
My child is always asking for a drink				**.635**					DD
If given the chance, my child would drink continuously throughout the day				**.838**					DD
If given the chance, my child would always be having a drink				**.830**					DD
My child has a big appetite (R)			-691						SR
My child leaves food on his/her plate at the end of a meal			-.398		**.609**				SR
My child gets full before his/her meal is finished			-.407		**.607**				SR
My child gets full up easily			-.529		**.501**				SR
My child cannot eat a meal if s/he has had a snack just before					**.505**				SR
My child finishes his/her meal quickly (R)						**.797**			SE
My child eats slowly						**.753**			SE
My child takes more than 30 minutes to finish a meal						**.771**			SE
My child eats more and more slowly during the course of a meal					.655				SE
My child eats less when angry							**.782**		EUE
My child eats less when s/he is tired					.436		**.473**		EUE
My child eats less when upset							**.794**		EUE
My child eats more when she is happy							**.734**		EUE

My child refuses new foods at first								**.855**	FF
My child enjoys tasting new foods (R)								**.851**	FF
My child enjoys a wide variety of foods (R)			-.354					**.724**	FF
My child is difficult to please with meals			-.435				.359	**.404**	FF
My child is interested in tasting food s/he hasn't tasted before (R)								**.829**	FF
My child decides that s/he doesn't like a food, even without tasting it								**.699**	FF
Cronbach`s alpha	.776	.763[1]	.858	.810[2]	.765	.707[3]	.736	.883	
Mean score (SD)	2.36 (.71)	1.45 (.49)	4.04 (.62)	2.17 (.87)	2.81 (.60)	2.66 (.67)	3.28 (.80)	1.87 (.69)	

[1]Without item: My child eats more when s/he has nothing else to do
[2]Without item: My child is always asking for a drink
[3]Without item: My child eats more and more slowly during the course of a meal

[a] Principal component analysis (PCA) with Varimax normalized rotation were run on all the items. Questions with reversed scales were first reversely scored, and a factor loading cut-off of 0.3 was applied before running the factor analysis. The factor analysis of the original 35-items, 8-factor original CEBQ resulted in a 31-item 8-factor model in this study, with the same eight factors as in the original CEBQ. The 8-factor model accounted for 62% of the total variance. Cronbach´s α was computed for each of the eight subscales. To improve the internal reliability of the subscales, the item "My child eats more when s/he has nothing else to do" was excluded from the subscale *Emotional overeating*, the item "My child is always asking for a drink" was excluded from the subscale *Desire to drink* and the item "My child eats more and more slowly during the course of a meal" was excluded from the subscale *Slowness in eating.*
