# Supplementary material for: Evaluation of an eHealth intervention aiming to promote healthy food habits from infancy -the Norwegian randomized controlled trial Early Food for Future Health
Source: Int J Behav Nutr Phys Act. 2019 Jan 3;16:1. doi: 10.1186/s12966-018-0763-4 (PMC6318886; doi:10.1186/s12966-018-0763-4)
Supplement: Supplementary file 4 — Confirmatory factor analysis for the Infant Feeding Questionnaire; factor loadings for all items and Cronbach alpha scores for each factor structure. (PDF 194 kb) [file 12966_2018_763_MOESM4_ESM.pdf]

Supplemental Table 2: Factor loadings for all items of the Infant Feeding Questionnaire (IFQ) and Cronbach alpha scores for each factor structure (12 months)

**Items**	**Factors determined through factor analysis**					**Original scale**
	Factor 1<br>*Awareness of infant satiety and hunger cues*	Factor 2<br>*Using food to calm fussiness*	Factor 3<br>*Feeding on schedule*	Factor 4<br>*Concern about infant undereating and being underweight*	Factor 5<br>*Concern about infant overeating and being overweight*	
My child knows when she/he is hungry	,638					Factor 1
My child knows when she/he is full	,580					“
I know when my child is full	,768					“
I know when my child is hungry	,706					“
To make sure s/he don`t get fussy, do you feed her/him even if you don`t think s/he is hungry?		,751				Factor 2
Feeding my child is the best way to stop him/her being unsettled		,825				“
When your child gets upset, is feeding him/her the first thing you do?		,686				“
Do you only allow your child to feed at set times?			,807			Factor 3
Do you let your child feed whenever s/he wants to? (R)			,724			“
Do you worry that your child is not feeding enough?				,836		Factor 4
Is it a struggle to get your child to feed?				,825		“

I am worried that my child will become underweight				,765		“
If I don`t encourage my child to feed, then s/he will not eat enough				,731		“
Do you worry that your child is feeding too much?					,820	Factor 5
I am worried that my child will become overweight					,777	“
Do you get upset if your child feeds too much?					,806	“
**Cronbach`s alpha**	,64	,65	,46	,81	,72	
**Mean score (SD)**	4,11 ± 0,60	2,12 ± 0,69	3,04 ± 0,77	1,87 ± 0,79	1,54 ± 0,65	

Principal component analysis (PCA) with Varimax normalized rotation were run on all the items. Questions with reversed scales were first reversely scored, and a factor loading cut-off of 0.3 was applied before running the factor analysis. The factor analysis in this study resulted in the same five factors as in the modified IFQ, explaining 62% of the total variance (Additional file2: Table S2). Cronbach´s α was computed for each of the five subscales: *Feeding on a schedule* (2 items; e.g., Do you let your baby feed whenever s/he wants to?, Cronbach´s α=0.46), *Awareness of hunger and satiety cues* (4 items; e.g., I know when my baby is hungry, Cronbach´s α=0.64), *Concern about infant overeating and becoming overweight* (3 items; e.g., Do you worry that your baby is feeding too much?, Cronbach´s α=0.72), *Concern about infant under-eating and becoming underweight* (4 items; e.g., I am worried that my baby will become underweight, Cronbach´s α=0.81), and *Using food to calm* (3 items, e.g., Feeding my baby is the best way to stop him/her being unsettled, Cronbach´s α=0.65).
