# Supplementary material for: Evaluation of an eHealth intervention aiming to promote healthy food habits from infancy -the Norwegian randomized controlled trial Early Food for Future Health
Source: Int J Behav Nutr Phys Act. 2019 Jan 3;16:1. doi: 10.1186/s12966-018-0763-4 (PMC6318886; doi:10.1186/s12966-018-0763-4)
Supplement: Supplementary file 5 — Group comparisons of baseline characteristics between participants who retained in the study and those who were lost to follow-up, and between the control and intervention group within the group of participants lost to follow-up. (PDF 95 kb) [file 12966_2018_763_MOESM5_ESM.pdf]

Additional file 5:

Group comparison of baseline characteristics between participants who retained in the study and those who were lost to follow-up, and between the control and intervention group within the group of participants lost to follow-up.

**Variable**	**Follow-up vs. Lost to follow-up**		**Lost to follow-up; Control vs. Intervention group**	
	Completed 12 months Q N=455	Did not complete 12 months Q N=260	Control group N=138	Intervention group N=122
**Mother**				
Age (years)	30.8 ± 4.2	29.8 ± 4.4*	29.7 ± 4.3	30.0 ± 4.6
Norwegian as native language	93.0 (423)	91.5 (238)	92.8 (128)	90.2 (110)
First-time mother (for infant participating in the survey)	58.5 (266)	54.2 (141)	55.1 (76)	53.3 (65)
Marital status				
Married	40.7 (185)	37.7 (98)	36.2 (50)	39.3 (48)
Cohabitant	56.9 (259)	60.8 (158)	61.6 (85)	59.8 (73)
Not married/cohabitant	2.4 (11)	1.5 (4)	2.2 (3)	0.8 (1)
Education (College/university degree)	84.2 (378)	76.9 (200)**	74.6 (103)	79.5 (97)
Main activity (before pregnancy)				
Working fulltime	81.6 (368)	78.8 (204)	78.8 (108)	78.7 (96)
Working part time	6.0 (27)	8.1 (21)	9.5 (13)	6.6 (8)
Student	6.9 (31)	8.1 (21)	5.1 (7)	11.5 (14)
Not working	5.5 (25)	5.0 (13)	6.6 (9)	3.3 (4)
BMI (kg/m^2)	24.9 ± 4.4	24.9 ± 4.2	24.5 ± 4.4	25.3 ± 4.0
Smoking	4.0 (18)	3.5 (9)	2.2 (3)	4.9 (6)
Use of snus	4.8 (22)	6.5 (17)	5.1 (7)	8.2 (10)
**Infant**				
Gender (female)	49.5 (225)	48.8 (127)	47.8 (66)	50.0 (61)
Gestational age>38 weeks	91.9 (418)	88.1 (229)	84.8 (117)	91.8 (112)
Birth weight (g)	3589 ± 492	3566 ± 492	3522 ± 490	3616 ± 493
Exclusive breastfed first month	66.8 (304)	68.1 (177)	71.0 (98)	64.8 (79)
Introduced to solid food before four months of age	5.1 (23)	4.2 (11)	2.2 (3)	6.6 (8)

* p=0.008, **p=0.016
